# The vertical structure of upper ocean variability at the Porcupine Abyssal Plain during 2012–2013

**DOI:** 10.1002/2015JC011423

**Published:** 2016-05-13

**Authors:** Gillian M. Damerell, Karen J. Heywood, Andrew F. Thompson, Umberto Binetti, Jan Kaiser

**Affiliations:** ^1^ Centre for Ocean and Atmospheric Sciences, School of Environmental SciencesUniversity of East AngliaNorwichUK; ^2^ Division of Geological and Planetary Sciences, California Institute of TechnologyPasadenaCaliforniaUSA

**Keywords:** intraseasonal variability, North Atlantic, ocean gliders

## Abstract

This study presents the characterization of variability in temperature, salinity and oxygen concentration, including the vertical structure of the variability, in the upper 1000 m of the ocean over a full year in the northeast Atlantic. Continuously profiling ocean gliders with vertical resolution between 0.5 and 1 m provide more information on temporal variability throughout the water column than time series from moorings with sensors at a limited number of fixed depths. The heat, salt and dissolved oxygen content are quantified at each depth. While the near surface heat content is consistent with the net surface heat flux, heat content of the deeper layers is driven by gyre‐scale water mass changes. Below ∼150m, heat and salt content display intraseasonal variability which has not been resolved by previous studies. A mode‐1 baroclinic internal tide is detected as a peak in the power spectra of water mass properties. The depth of minimum variability is at ∼415m for both temperature and salinity, but this is a depth of high variability for oxygen concentration. The deep variability is dominated by the intermittent appearance of Mediterranean Water, which shows evidence of filamentation. Susceptibility to salt fingering occurs throughout much of the water column for much of the year. Between about 700–900 m, the water column is susceptible to diffusive layering, particularly when Mediterranean Water is present. This unique ability to resolve both high vertical and temporal variability highlights the importance of intraseasonal variability in upper ocean heat and salt content, variations that may be aliased by traditional observing techniques.

## Key Points

- Intraseasonal variability in upper ocean temperature and salinity, not previously reported
- Mode‐1 baroclinic internal tide detected
- Deep variability dominated by intermittent patches of Mediterranean Water, which show evidence of filamentation

## 1 Introduction

The ocean and the atmosphere exchange heat, salt, momentum and tracers through an ocean surface boundary layer, in which biological activity is also focused. However, long time series of upper ocean observations are challenging to obtain. Most previous studies of upper ocean variability have relied on ship CTD profiles (Conductivity, Temperature, Depth) with limited temporal coverage and resolution and an inevitable summer bias (examples in the northeast Atlantic include *Bray* [1982]; *Harvey* [1982]; *Rios et al*. [1992]; *Prieto et al*. [2013]) or on moorings with instruments at a limited number of depths [*Chidichimo et al*., 2010; *Machin et al*., 2010; *Hartman et al*., 2012]. Even studies which combine ships, moorings, Argo floats and satellite observations [*Hartman et al*., 2010; *Ullgren and White*, 2010, 2012], do not obtain coverage of a full year with sufficient temporal and vertical resolution to capture many ocean processes.

**Figure 1:**
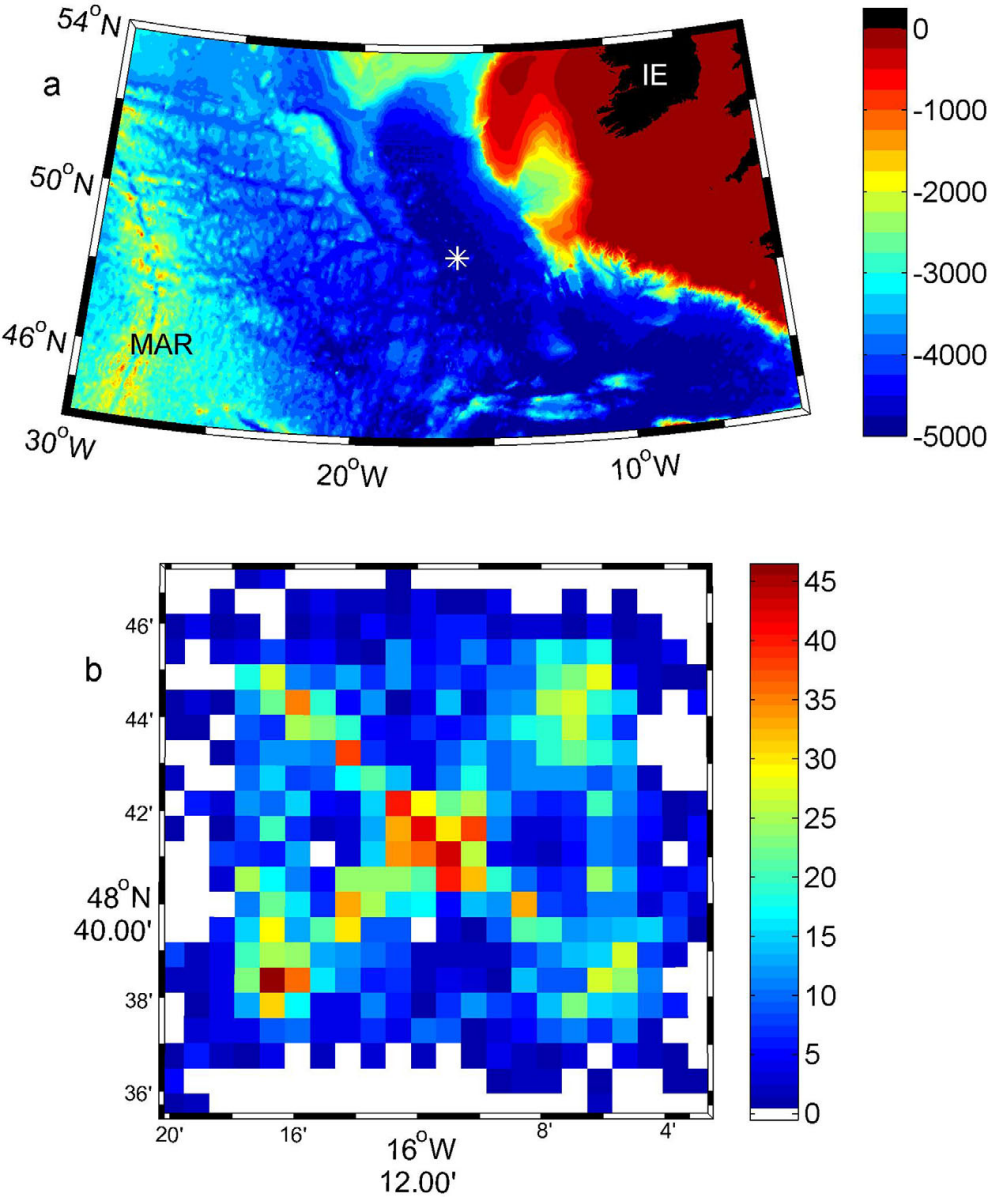
(a) Bathymetry of the north‐east Atlantic basin. The white asterisk marks the location of the OSMOSIS field campaign. MAR=mid‐Atlantic Ridge. IE=Ireland. (b) Number of profiles in each 1 × 1 km grid cell collected during the OSMOSIS campaign and used in this paper (i.e., from only one Seaglider at a time, not both), centred around 48.7°N, 16.2°W.

Here we document an ocean glider‐based study of the temporal variability of the upper ocean. The Ocean Surface Mixing, Ocean Submesoscale Interaction Study (OSMOSIS) incorporated a year‐long observational program centered 41 km to the southeast of the Porcupine Abyssal Plain sustained observatory (PAP‐SO), with observations collected within a 15 km radius of 48.7° N, 16.2° W (Figure 1). The PAP‐SO [*Lampitt et al*., 2010] is situated in the Northeast Atlantic (49.0° N 16.5° W) at a water depth of 4800 m. This location is considered remote from the topographic complexities of the continental slope and the Mid‐Atlantic Ridge [*Hartman et al*., 2012], and thus remote from places where strong internal tides might be generated. It is located in the inter‐gyre region between the North Atlantic subpolar and subtropical gyres where the mean flow is relatively weak and eddy kinetic energy is moderate. The variability in physical properties is likely to be representative of large areas of the mid‐latitude gyres.

As part of the OSMOSIS field campaign, pairs of Seagliders were deployed for periods varying between two and 5 months, between them covering an entire year from 4 September 2012 to 7 September 2013. Here we use the glider data set to show the seasonal evolution of the uppermost 1000 m of the water column and determine the characteristic timescales of variability in temperature, salinity and dissolved oxygen concentration. We discuss the likely causes of that variability and how it compares with surface forcing such as the heat and freshwater fluxes, and wind stress. This allows us to resolve fluctuations in processes whose signature in vertical variability happens on relatively small scales, such as the internal tides, and compact mesoscale features.

## 2 Data and Methods

The Seaglider is a small, autonomous, buoyancy‐driven vehicle which profiles to a maximum depth of 1000 m in a sawtooth pattern [*Eriksen et al*., 2001]. All the Seagliders deployed during the OSMOSIS field campaign carried a Seabird SBE3 temperature sensor and SBE4 conductivity sensor (known collectively as the CT sail), and an Aanderaa 4330F oxygen optode. Following calibration (see below), temperature, salinity and oxygen concentrations are accurate to 0.01°C, 0.01 g kg^−1^ and 2 *μ*mol kg^−1^, respectively. Sensor precision is 0.001°C and 0.0003 S m^−1^ for temperature and conductivity respectively, combining to a salinity precision of approximately 0.001 g kg^−1^. Sampling occurred approximately every 5 s (0.5 m vertical resolution at typical vertical speeds of 0.1 m s^−1^) in the upper part of the water column, and every 10 s (1 m vertical resolution) below that. The depth at which the vertical resolution changed varied between 200 and 400 m, depending on battery constraints.

The Seaglider hydrodynamic flight model is tuned following *Frajka‐Williams et al*. [2011]. Dive‐average currents are calculated from the difference between the glider's flight path found from GPS positions at the beginning and end of each dive, and the glider's flight path as calculated from the Seaglider hydrodynamic model. The thermal lag of the CT sensor is corrected following the methods of *Garau et al*. [2011]. Occasional poor quality data (e.g., from biofouling of the conductivity sensor, from poor flushing of the conductivity cell when the glider is moving slowly) are flagged and discarded; this accounts for 2.6% of the total data collected. CTD casts were collected from the ships *RRS Discovery* (September 2012), *RV Celtic Explorer* (January 2013), and *RRS James Cook* (April, June and September 2013). Salinity and dissolved oxygen concentrations from the Seagliders were calibrated against the ship CTD salinities and dissolved oxygen concentrations from each cruise, which in turn were calibrated against discrete water samples analyzed with an Autosal salinometer and an automated Winkler titration system.

**Figure 2:**
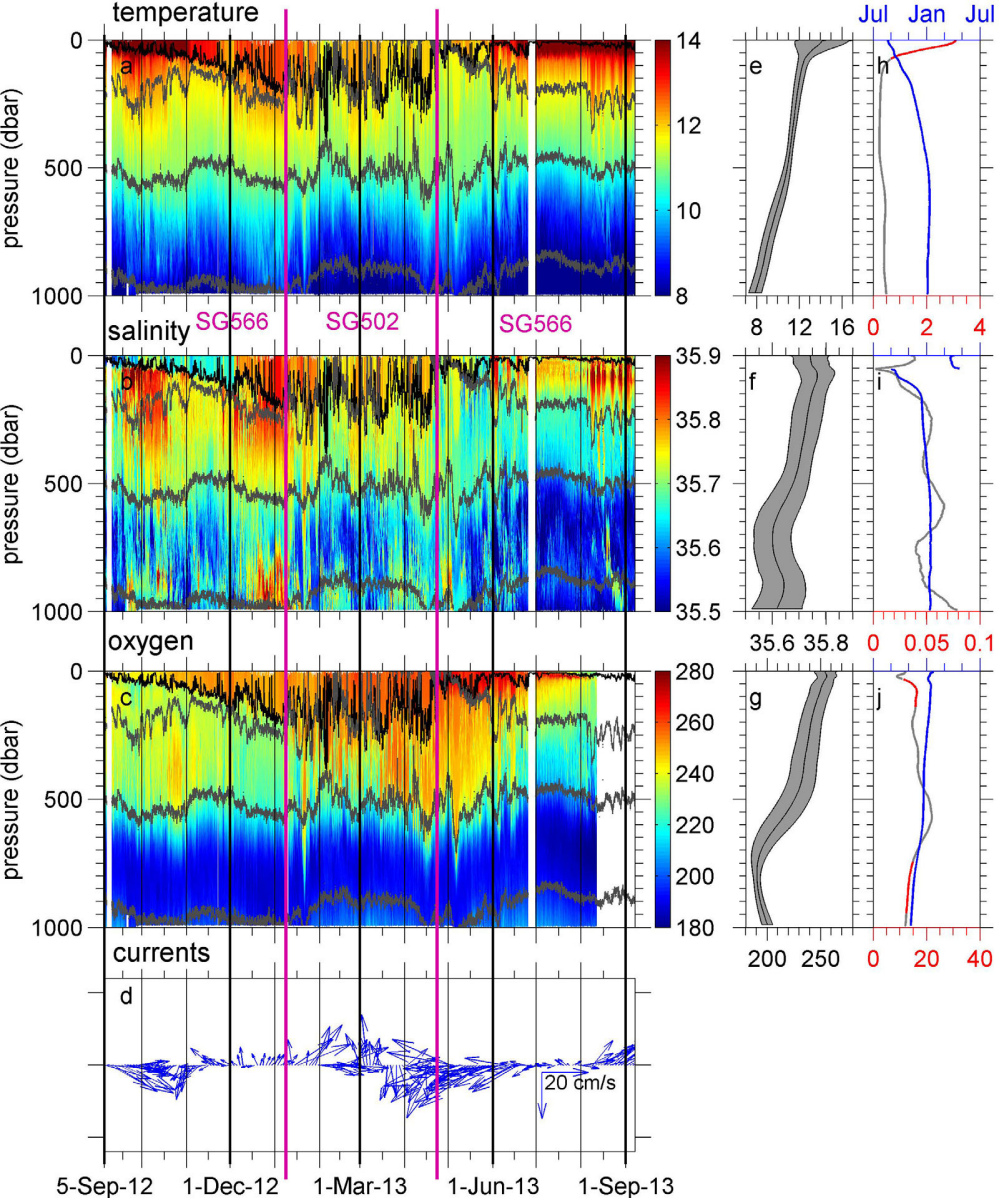
Time series of (a) conservative temperature (°C), (b) absolute salinity (g kg^−1^), (c) dissolved oxygen concentration (*μ*mol kg^−1^), and (d) dive‐average currents (cm s^−1^, detided), as measured by gliders SG566 (September–January), SG502 (January–April) and SG566 (April–September). Different gliders are separated by pink vertical lines, and black vertical lines are gridlines every month, with thicker black lines every 3 months to show approximate seasons. In Figures 2a–2c the black contour shows the ML depth and the grey contours show $\sigma_{\theta }$ surfaces 27.04, 27.2 and 27.6. The dive‐average currents are shown as 2 day averages for clarity. (e) The mean temperature (central line) plus and minus one standard deviation (grey area). (f and g) Are as Figure 2e for salinity and dissolved oxygen concentration respectively. (h–j) The phase (blue) and amplitude of the annual harmonic at each depth for temperature, salinity and dissolved oxygen concentration respectively. The amplitude is shown in red when the annual harmonic is a good fit to the observations, grey otherwise (see main text). The phase is represented as the time of year of the peak of the annual harmonic.

Figure 1b) shows the observational density of the glider profiles used in this paper. These are taken from one glider during each deployment period, selecting the glider which remained most closely within the OSMOSIS observational domain, and which had the least sensor issues (e.g., minimal biofouling of the conductivity cell). By concatenating three glider deployments we obtain a time series for the entire year totaling 4096 profiles (Figure 2). (The OSMOSIS observational programme collected 8138 glider profiles in total, but the data from the second glider during each deployment are not used in the time series analysis conducted here.) 95% of the 4096 profiles used in this paper lie within 15 km of 48.7° N, 16.2° W. 15 km is comparable to the spacing between CTD locations of a typical ship‐based hydrographic survey, and for the purposes of this paper, we treat the data as if they had all been obtained at the same location. There is an implicit linkage between spatial and temporal variability in glider observations, and here we choose to treat it as purely temporal variability. Submesoscale motions and small‐scale spatial variability observed in this data set are discussed by *Thompson et al*. [2016]. Glider profiles collected outside the study region (i.e., more than 15 km from 48.7° N, 16.2° W) are not included, and after removal of these and the occasional poor quality data (as above), 3785 profiles remain.

**Figure 3:**
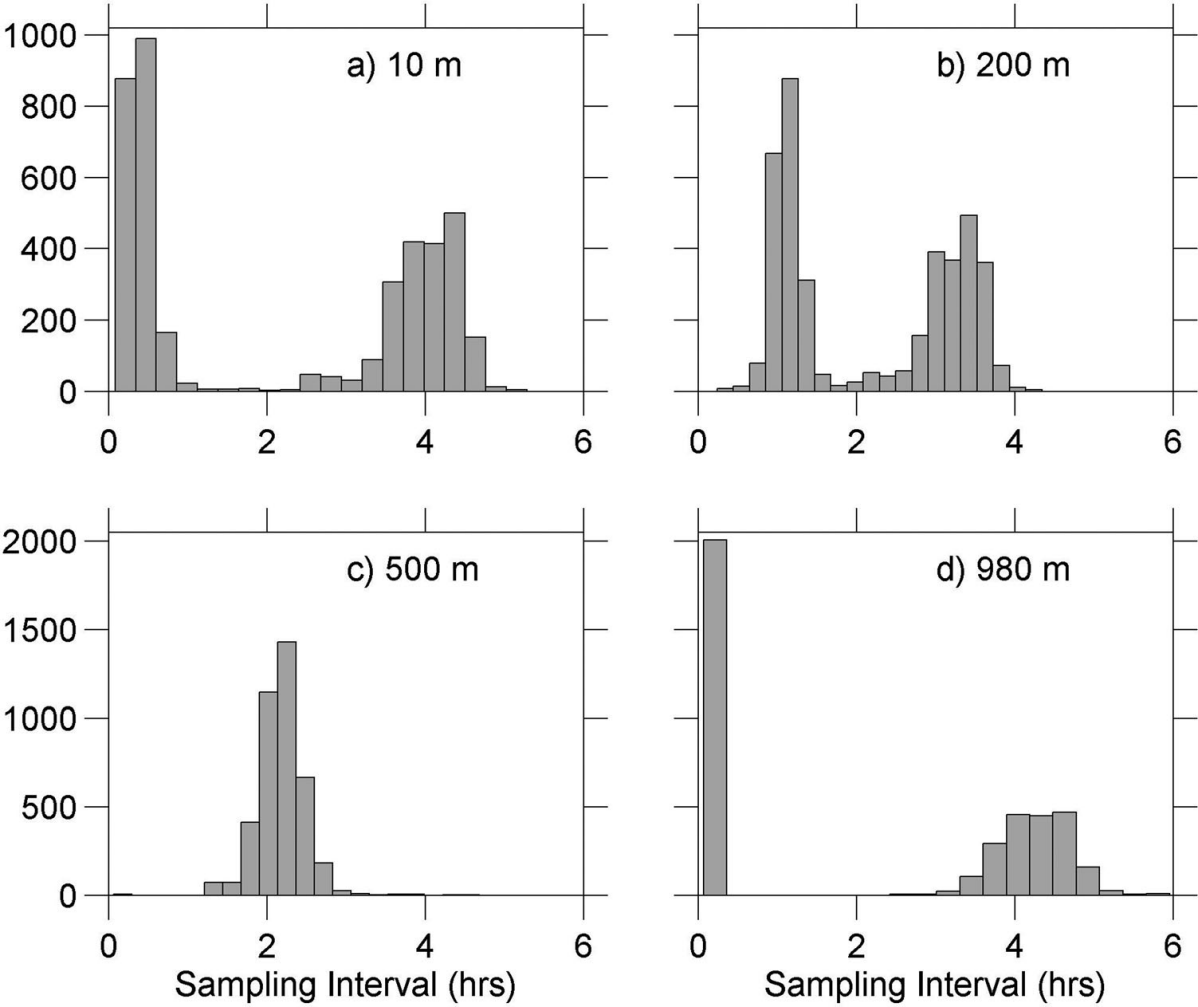
Histograms of the intervals between profiles at example depths: (a) 10 m, (b) 200 m, (c) 500 m, (d) 980 m.

To attribute variability to physical processes including mesoscale variability, wave motion and tides, we use the multitaper method [*Thomson*, 1982; *Percival and Walden*, 1993], to generate frequency spectra of temperature, salinity, dissolved oxygen concentration and dive‐average currents. The average dive duration was ∼4 h, and we treat the dive and climb sections of each glider dive as separate vertical profiles. Although samples along a constant depth surface are obtained at roughly 2 hourly intervals mid‐way down the profiles, at the surface and at dive‐apogee two profiles are obtained within a few minutes of each other followed by a near 4 h delay until the next two profiles are obtained (Figure 3). We therefore average the data into 4 h bins, giving a Nyquist frequency of 1 cycle per 8 h, or 3 cycles day^−1^, for the entire data set. The glider takes typically 6 days to occupy the survey pattern, occupying each corner of the domain in turn in a bow‐tie pattern, so any apparent 4–10 day signal may represent spatial variability, sampled by the glider as it moves through the survey box, that for the purposes of this analysis is interpreted solely in the time domain. There will also be some aliasing of internal waves. The buoyancy frequency, which represents the upper bound of the internal wave frequency band, varies in this data set from 0.001 to 0.05 s^−1^, corresponding to internal waves with periods of a few minutes (in the pycnocline) up to 2 h (in the weak stratification of the layer below the pycnocline down to ∼500 m). Variance at higher‐than‐resolved frequencies, including from internal waves (or, due to glider spatial sampling, at low frequencies but higher‐than‐resolved wavenumbers) will be aliased onto those resolved in this data set, resulting in a distortion of the observed spectra relative to the true values, particularly at higher frequencies [*Rudnick and Cole*, 2011]. Spectra computed from moored instruments deployed as part of OSMOSIS below the surface layer, sampling every 10 min (supporting information Figure S1), are very similar to those from the gliders, indicating that this effect does not significantly influence the conclusions here.

The depth of the surface mixed layer (ML) is calculated using a threshold value of temperature or density from a near‐surface value at 10 m depth ( ΔT=0.2°C or $\Delta \sigma_{\theta }=0.03$), whichever is the shallower [*de Boyer Montegut et al*., 2004]. Thus, we aim to find the depth of the ML even in cases where temperature and salinity vary with depth in a density‐compensating manner, as well as cases where density varies with depth due to changes in salinity rather than temperature. Annual harmonics of temperature, salinity and oxygen concentration are found by fitting (using a least squares approach) a sine wave with a period of 365 days to the temperature, salinity and oxygen concentration at each depth: the amplitude and phase of the fitted sine waves are shown in Figures 2h–2j. To highlight whether the annual harmonic is a good fit to the observations at each depth, we divide the standard deviation of the residuals (observations minus fitted sine wave) by the standard deviation of the observations. When this “goodness‐of‐fit” number (*γ*) is small, the residuals are small and much of the variance in the observations is explained by an annual harmonic. For illustrative purposes, Figure 2h‐j colors the amplitude red when *γ* is less than 0.6 (40% of variance explained by annual harmonic), but this is not intended to denote an abrupt cut‐off from good fits to bad. Using γ=0.5 (0.7), for example, simply contracts (expands) the depths colored red by ∼50 m.

Vertical diffusive and gravitational stability are assessed from individual profiles by calculating the spiciness *π* and stability angle ϕ following *Flament* [2002]. Spiciness is a state variable used to characterize water masses, being largest for warm and salty waters. ϕ is calculated over vertical intervals larger than the scale of the fine‐structure, here found to be ∼50 m. In this calculation it is assumed that variability over these vertical scales is of sufficiently low vertical‐to‐horizontal aspect ratio that it is not appreciably distorted by the slantwise profiling of the Seaglider. |ϕ|>90° indicates that the water column is gravitationally unstable, |ϕ|<45° indicates that the water column is diffusively stable, diffusive layering occurs when 45°<ϕ<90° and salt fingering when −90°<ϕ<−45°.

Sea surface temperature (SST), surface wind speed and surface fluxes of heat (shortwave and longwave radiation, latent and sensible heat fluxes) and freshwater (precipitation and evaporation) were extracted from the European Centre for Medium Range Weather Forecasting (ECMWF) ERA‐Interim reanalysis [*Dee et al*., 2011] at the nearest gridpoint to the OSMOSIS site (24 km west of the center of the OSMOSIS site). (The PAP‐SO meteorological buoy failed for approximately 6 months of the OSMOSIS observational period and it was considered better to use a consistent source for meteorological variables for the whole year.) A hypothetical ML temperature was derived by assuming the net surface heat flux is the only source of temperature change in the ML. It was calculated as follows:

(1) $$\Delta T=\frac{F\Delta t}{c\rho h}$$

where ΔT is the temperature change over a time period Δt (here 6 h, the time step of the ERA‐Interim reanalysis data set), *F* is the surface heat flux, *ρ* the ML density, and *h* the ML depth at that time. *c* is the specific heat capacity of seawater appropriate when using conservative temperature [*IOC*, *SCOR, and IAPSO*, 2010]. This gives a temperature change rather than an absolute temperature; for ease of display we set the hypothetical ML temperature equal to the observed ML temperature at the coldest point in the year. Similarly, a hypothetical ML salinity was calculated from the ERA‐Interim net surface freshwater flux as follows:

(2) $$S_{n}=S_{0}\prod_{t=1}^{n}\left(\frac{h_{t}}{h_{t}+FW_{t}.\Delta t}\right)$$

where *S_n_* is the salinity at time t=n.Δt, *S*_0_ is an initial salinity at time *t* = 0, *h_t_* is the ML depth at time *t* and *FW_t_* is the net freshwater flux at time *t*. The evolution of this hypothetical mixed layer salinity is not sensitive to the choice of initial value, *S*_0_.

Note that throughout this paper we use conservative temperature and absolute salinity (*S_A_*) following *IOC, SCOR, and IAPSO* [2010]. All densities are potential density anomalies ( $\sigma_{\theta }$) relative to the surface and will be given without units.

## 3 Results and Discussion

### 3.1 Water Masses and Vertical Stability

**Figure 4:**
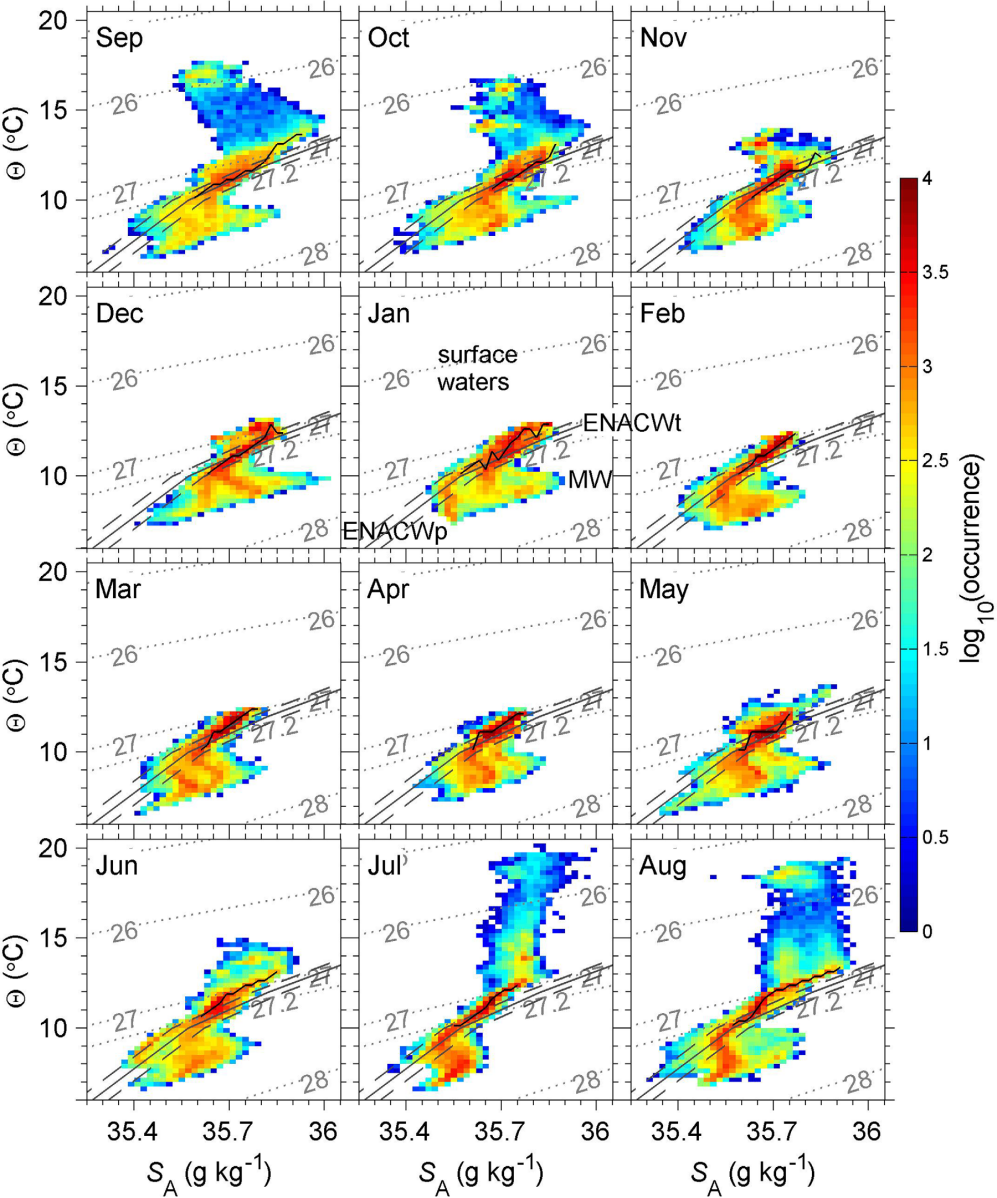
$\Theta -S_{A}$ diagrams for each month. The colors show the log_10_ of the number of data points in that $\Theta -S_{A}$ grid cell (i.e., the occurrence). The dotted light grey lines are $\sigma_{\theta }$ isopycnals. The $\sigma_{\theta }=27.2$ isopycnal is included to illustrate the boundary between ENACWt and ENACWp. The solid, darker grey line denotes ENACW as defined by *Harvey* [1982] and extended by *Rios et al*. [1992], with ENACWp at the cooler and fresher end, and ENACWt at the warmer and saltier end. Water mass labels are included on the January plot. Points between the grey dashed lines ( $S_{ENACW}\pm 0.05$) can be considered as ENACW. (We have converted the ENACW line to conservative temperature and absolute salinity.) The black line joins points of maximum occurrence for each isohaline, referred to in the main text as the “core” of the water masses observed here. It is only shown for $\Theta -S_{A}$ grid cells containing more than 500 data points, and for temperatures >10°C.

The frequency of occurrence of the main water masses observed throughout the year is illustrated in Figure 4. Surface waters ( $\sigma_{\theta }<27$) are the warmest water masses (11–20°C) and highest in dissolved oxygen concentration (Figure 2c). The $\sigma_{\theta }=27$ isopycnal reaches its maximum depth of approximately 200 m in July (Figure 2). The subsurface fresher, colder and less oxygenated water masses are Eastern North Atlantic Central Water (ENACW) of subtropical (ENACWt) and subpolar (ENACWp) origin [*Harvey*, 1982]. ENACW is thought to be formed by deep winter mixing in a wide region from the Azores to the European boundary, bounded on the west and north by the North Atlantic Current and to the south by the Azores Current [*Pollard and Pu*, 1985; *Pollard et al*., 1996]. ENACWt is found at $\sigma_{\theta }$ in the range 27–27.2, and is warmer, saltier and more oxygenated than ENACWp. In this data set, ENACWt extends down to approximately 500 m (as seen from the depth of the 27.2 isopycnal on Figure 2), below which we find ENACWp. The slight salinity minimum around $\sigma_{\theta }=27.3$ is characteristic of the influence of Sub‐Arctic Intermediate Water [*Arhan*, 1990]. Previous studies in this region have shown that waters which are slightly fresher than ENACW at similar densities show some mixing with Western North Atlantic Water (WNAW). Specifically, water with a $\Theta -S_{A}$ relationship parallel with the ENACW line but fresher by 0.1 at the same temperature can be considered WNAW [*Rios et al*., 1992; *Pollard et al*., 1996].

The “core” of the water masses observed here (Figure 4) is defined as a line joining the grid cell maximum occurrence for each isohaline. The water mass core is only shown for temperatures >10°C as below this the water mass properties are significantly influenced by the presence of Mediterranean Water (MW) so comparisons with ENACW are not relevant. The core lies, at times, along the line of ENACW, but is often shifted somewhat to the left of this line (especially in summer), i.e., the waters are warmer and/or fresher. If this shift were entirely due to a change in temperature, then on average the core observed here is 0.40°C warmer than the ENACW line at the same salinity. The shift might be due to a difference in salinity rather than temperature, but the maximum salinity difference along isotherms between the core observed here and the ENACW line reaches 0.16 in September, and is greater than 0.1 in 9 months out of 12 (Figure 4). In other words, if the difference were purely due to a change in salinity then the waters observed here, well within the eastern North Atlantic, are even fresher than WNAW. Thus we posit that at least some of the difference between the water mass core observed here and the ENACW line must be due to an increase in temperature since the ENACW line was first defined by *Harvey* [1982], using data collected in the 1950s and 1960s.

The oxygen minimum layer occurs at an average depth of 785 m. Below 700 m, there are intermittent patches of high salinity due to the influence of MW [*Mauritzen et al*., 2001], characterized by a salinity maximum (up to 36.01 g kg^−1^) centered at $\sigma_{\theta }=27.6$ (Figure 4). These MW characteristics are similar to those observed by *Ullgren and White* [2010, 2012] at the southern entrance to the Rockall Trough, 480 km north of the OSMOSIS site. ENACWp and MW are both found at $\sigma_{\theta }>27.2$ (Figures 2 and 4). There is more MW in winter than in summer with an especially noticeable patch of very saline MW from mid‐December to mid‐January (Figure 2b), consistent with the results of *Prieto et al*. [2013], who find that MW detaches from the Iberian slope and spreads into the outer ocean more in winter than in summer. Below the MW, there is perhaps some evidence of the colder and fresher Labrador Sea Water, but this data set does not extend deep enough to explore that fully.

**Figure 5:**
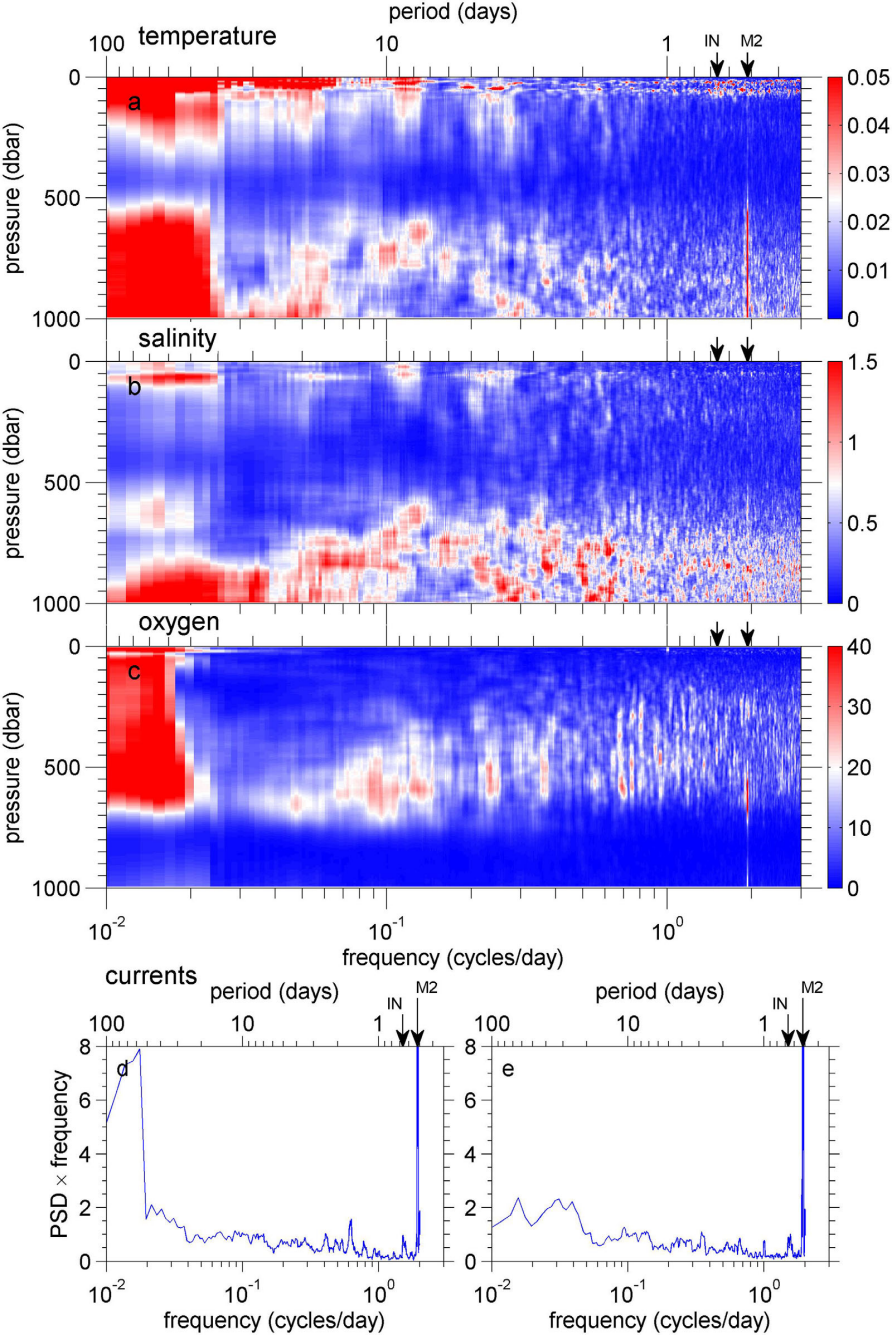
Variance preserving spectra for (a) temperature (b) salinity, (c) dissolved oxygen concentration, (d) zonal, and (e) meridional component of the dive‐average currents, all $\times 10^{-3}$ except temperature and dissolved oxygen. In Figures 5a–5c, the colors show the power spectral density × frequency. The inertial frequency (IN) and M2 tidal frequency are marked as black arrows on the upper axis.

**Figure 6:**
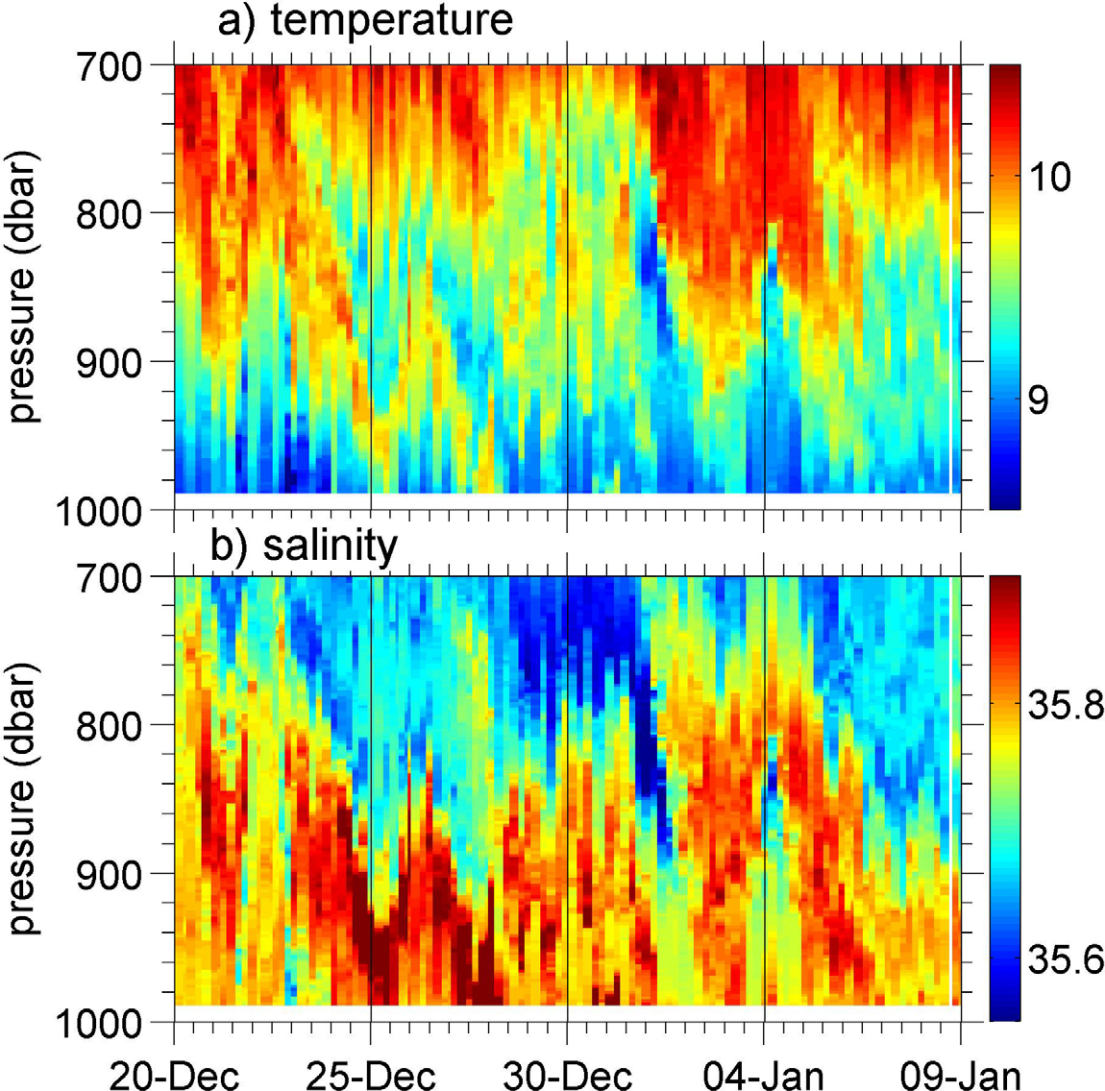
Expanded view of (a) conservative temperature (°C) and (b) absolute salinity (g kg^−1^) of a patch of Mediterranean Water showing the filamented nature of the patch, with high variability in time and depth.

Depths between approximately 700–1000 m exhibit high variability at all timescales in salinity and temperature but much less so in dissolved oxygen concentration (Figure 5). The high variability in salinity and temperature at these depths can be ascribed to the occurrence of MW being intermittent on multiple timescales. The patches of MW show variability on time scales as short as a day, and *Thompson et al*. [2016] discuss their spatial variability across the ∼20 km OSMOSIS domain. These features are suggestive of a filamentary structure (an example is shown in Figure 6). However, the ENACWp and MW have similar dissolved oxygen concentrations (∼194 and ∼ 189 *μ*mol kg^−1^ respectively), so the oxygen concentration does not exhibit as much variability as temperature and salinity (Figure 5).

**Figure 7:**
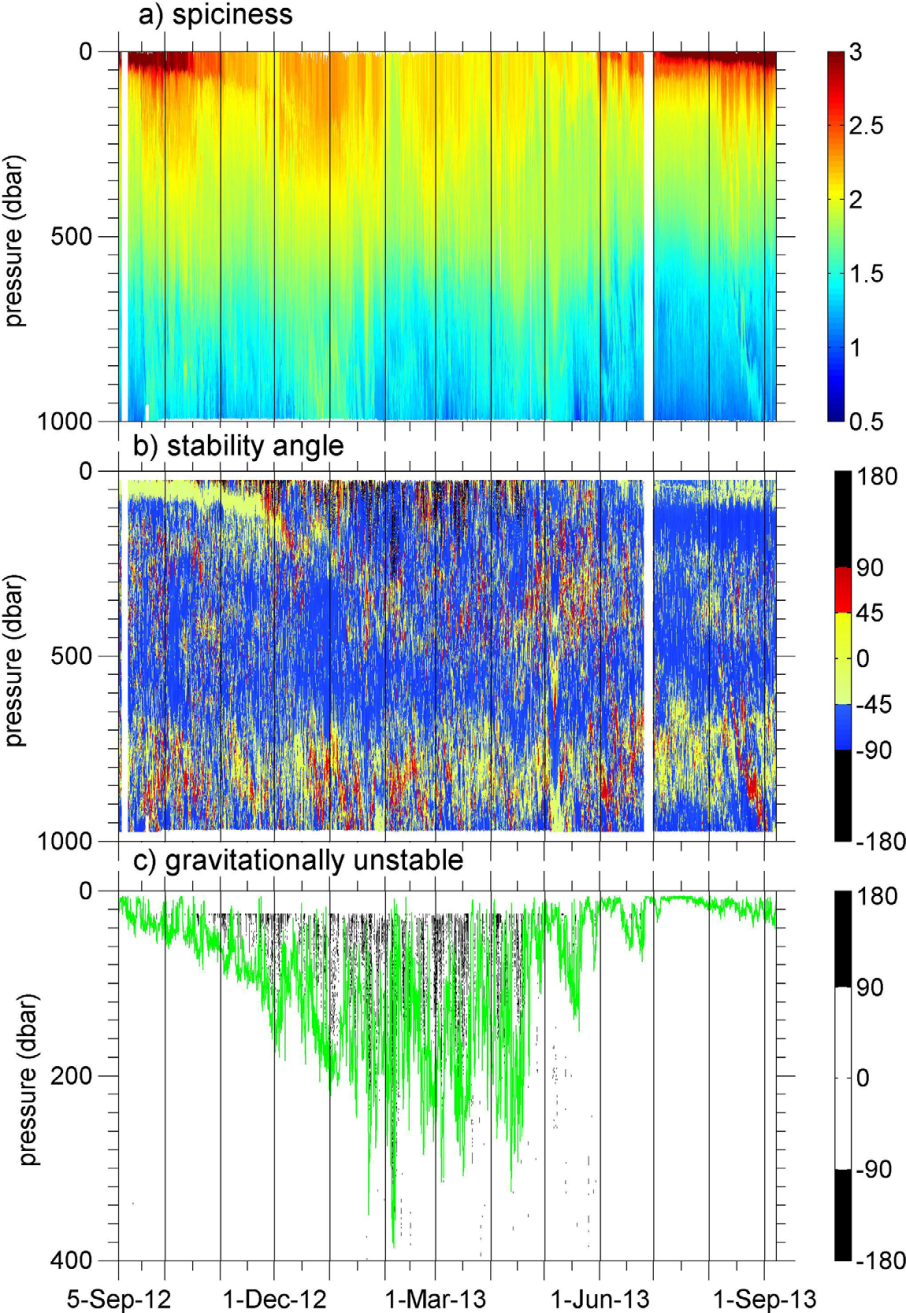
Time series of (a) spiciness (*π*) and (b) stability angle ( ϕ). |ϕ|>90° indicates that the water column is gravitationally unstable (black), |ϕ|<45° indicates that the water column is diffusively stable (yellow), diffusive layering occurs when 45°<ϕ<90° (red) and salt fingering when −90°<ϕ<−45° (blue). Note that because ϕ is calculated over 50 m, there are no ϕ values in the top and bottom 25 m. (c) An expanded view of ϕ above 400 m, showing only those occasions when it is gravitationally unstable. In Figure 7c, the green line is the ML depth.

The MW/ENACWp layer between approximately 700 and 1000 m is often diffusively stable (Figure 7), but with periods of susceptibility to diffusive layering largely corresponding to times when more saline MW is present (Figure 2b). ENACWt is largely susceptible to salt fingering, but between December and April when ENACWt extends to the surface there are occasional periods when the ML is gravitationally unstable (Figure 7c), which correspond to rapidly deepening ML depths due to convective overturning. The gravitational instabilities observed here are of comparable magnitude to those observed by *Anis and Moum* [1992], consisting of perturbations from a stable profile of order 0.01–0.02°C (considerably larger than the sensor precision). We are observing ENACW as it is being locally formed by deep winter mixing. Since the stability angle ϕ is calculated over vertical intervals of 50 m, there appear to be no gravitational instabilities shallower than 25 m in Figure 7c. This leads to an inevitable bias toward detecting gravitational instabilities in the ML in winter, rather than in summer when the ML is shallow. However, in Figure 4 one can also see the $\Theta -S_{A}$ properties collapsing onto the ENACW line toward the end of winter (except at greater densities where some MW influence remains), whereas in summer the water mass properties at the surface diverge, which would not be the case if there was frequent convective overturning due to gravitational instabilities.

### 3.2 Mixed Layer Variability and Air Sea Fluxes/Exchange

**Figure 8:**
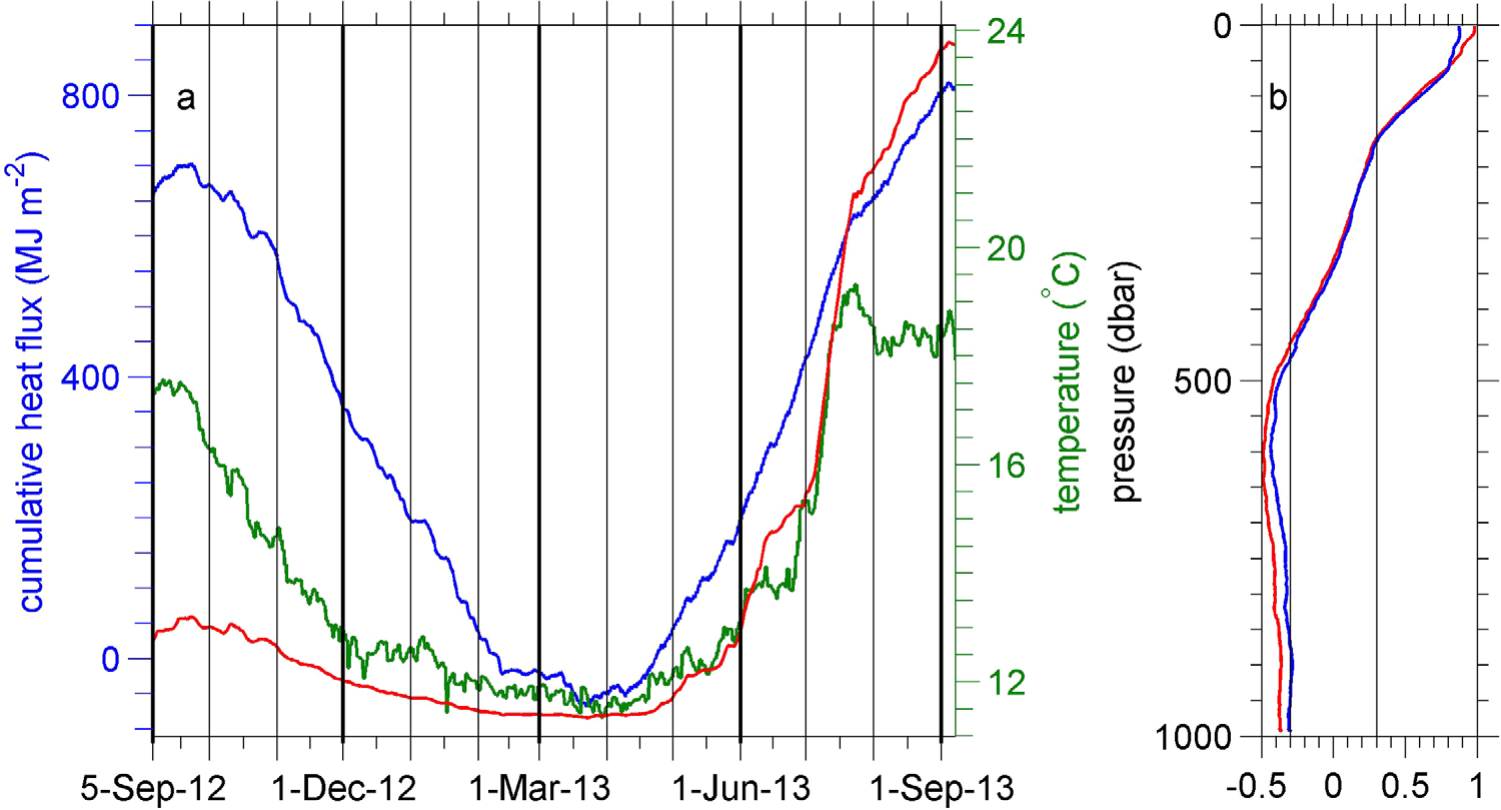
(a) Time series of cumulative net surface heat flux (MJ m^−2^, positive downward, blue), sea surface temperature (SST, °C, green) from the ECMWF ERA‐Interim reanalysis data set at the nearest gridpoint to the OSMOSIS site, and a hypothetical ML temperature calculated by assuming the surface heat flux is the only source/sink of heat in the ML (red, see main text). (b) The correlation with depth between the glider‐measured temperature and the cumulative net surface heat flux (blue) and SST (red) at zero lag. Correlations with magnitude > 0.3 are considered to be significant: the black vertical lines mark correlations of −0.3 and + 0.3.

The obvious seasonal cycle in the temperature of the uppermost 150 m is due to solar insolation (Figures 2a and 8). Temperatures within the top 10 m, where the amplitude of the annual harmonic is greatest (Figure 2h), range from approximately 12°C in winter to 19°C in July, comparable to that observed by *Hartman et al*. [2010] at the PAP‐SO between 2003 and 2005. The temporal standard deviation of temperature (Figure 2e) decreases rapidly from the surface to approximately 60 m and decreases slowly to ∼200 m. The minimum in standard deviation of temperature occurs at ∼400 m. Below 150 m an annual harmonic does not fit the observed variability well.

The ML temperature is strongly correlated (*r* = 0.98, Figure 8b) with the ECMWF ERA‐Interim SST. The ML temperature is also correlated with the cumulative ERA‐Interim net surface heat flux into the ocean (*r* = 0.87). (Here the ML temperature is averaged to the same times as the ERA‐Interim data.) Temperatures below 150 m are not correlated with the cumulative net surface heat flux (Figure 8b). Figure 8a also shows (red curve) the hypothetical ML temperature derived by assuming that the surface heat flux is the only source of temperature change in the ML. This hypothetical ML temperature will only be reasonable if there is no heat flux from/to the ocean interior (whether by entrainment or by the diffusive export of heat from the ML to the layer below such as discussed by *Cronin et al*. [2015] and *Lee et al*. [2015]), no advection of water with anomalous temperatures by the circulation, no horizontal or vertical mixing with waters at a different temperature, even when the depth of the ML increases, and if all the surface heat flux is absorbed in the ML. This hypothetical ML temperature covaries with the actual ML temperature reasonably well during the winter and during the warming in spring (within 1°C from the start of December to the end of May), but not in autumn when the ML deepens and cools, and also not during late summer. In late summer, the ML is often very shallow (see below and Figure 2) and it is likely that some fraction of the solar absorption occurs below the ML due to penetrative radiation. Thus the hypothetical ML temperature should be considered as, at best, an upper bound on the possible ML temperature tendency at times when the ML is very shallow. Cooling occurs primarily at times when the ML is deepening and there is a temperature difference between the ML and waters below (Figures 2a and 8a). This cooling of the ML may be initiated by cooling at the surface leading to convective overturning, but the subsequent change in temperature is also influenced by entrainment of cooler waters from the ocean interior, such as are seen just below the ML during the autumn (Figure 2a).

The ML salinity does not have an obvious seasonal cycle, is not well described by an annual harmonic, and is variable on all time scales (Figure 5). It is not correlated with the Aquarius satellite sea surface salinity (Level 3 Sea Surface Salinity Standard Mapped Image 7 Day Data V3 [*Lee et al*., 2012]) at the nearest gridpoint to the OSMOSIS site. The hypothetical ML salinity expected from the ERA‐Interim surface freshwater flux (not shown) is not correlated with the measured salinity, and the range is an order of magnitude smaller than that of the measured salinity. Thus precipitation and evaporation are not the major drivers of changes in salinity in the ML. The changes in ML salinity must be primarily due to advection into the area of water masses of different salinity and/or vertical mixing with waters of different salinity from the ocean interior. The changes are too persistent to be solely due to eddies and are therefore likely to be associated with variations in the gyre‐scale circulation.

The de‐tided dive‐average currents (Figure 2d) are weak, reaching a maximum of 0.38 m s^−1^ in late January. In only 1/4 of the record is the speed above 0.2 m s^−1^. *Painter et al*. [2010] found velocities of a comparable magnitude during a vessel‐mounted Acoustic Doppler Current Profiler survey at the PAP‐SO in 2006. Despite the low speeds, the dive‐average currents are often persistent in direction for periods of a month or more. During September 2012 and January, February and August 2013, the currents are persistently eastward. From mid‐April to late June, the currents are persistently westward. The dive‐average currents are not correlated with local ECMWF ERA‐Interim wind speeds, but they are weakly correlated with the salinity ( r≈0.4) observed by the gliders down to 300 m (not shown), suggesting that the advection of different water masses into the region is one source of the water mass variability. Mixed layer dissolved oxygen concentration is dominated by the temperature dependence of oxygen solubility [*Emerson*, 1987; *Najjar and Keeling*, 1997], biological processes and air‐sea gas exchange and is not discussed further in this paper.

The ML depth observed here is comparable to that discussed by *Hartman et al*. [2010], which was obtained by taking monthly averages of the ML depth from Argo float data collected in a region centred around PAP‐SO (45°N to 52°N, 26.08°W to 8.92°W, excluding the shelf area) between January 2003 and July 2005. *Hartman et al*. [2010] also found that the ML depth reaches a maximum of approximately 300–400 m (though generally in March whereas here the maximum depth occurred in early February), and that the ML depth has much greater variability in winter than in summer. We find shallower ML depths in the summer than they observed: in July and August 288 out of 654 profiles show stratification up to the minimum depth (3–5 m) reliably observed by the gliders, whereas the minimum ML depths observed by *Hartman et al*. [2010] were approximately 20–30 m. This difference is likely due to the higher vertical resolution of the Seagliders compared with Argo floats. The glider campaign provides greater temporal resolution, allowing us to observe, for example, that the spring shoaling of the ML is not a gradual and smooth process; instead there is a rapid onset of shoaling in April (daily average ML depths change from being around 200 m to around 50 m in 2 days) followed by several deepening and restratifying events in May and June (seen most clearly in Figure 7c).

### 3.3 Intraseasonal Variability Below the Mixed Layer

**Figure 9:**
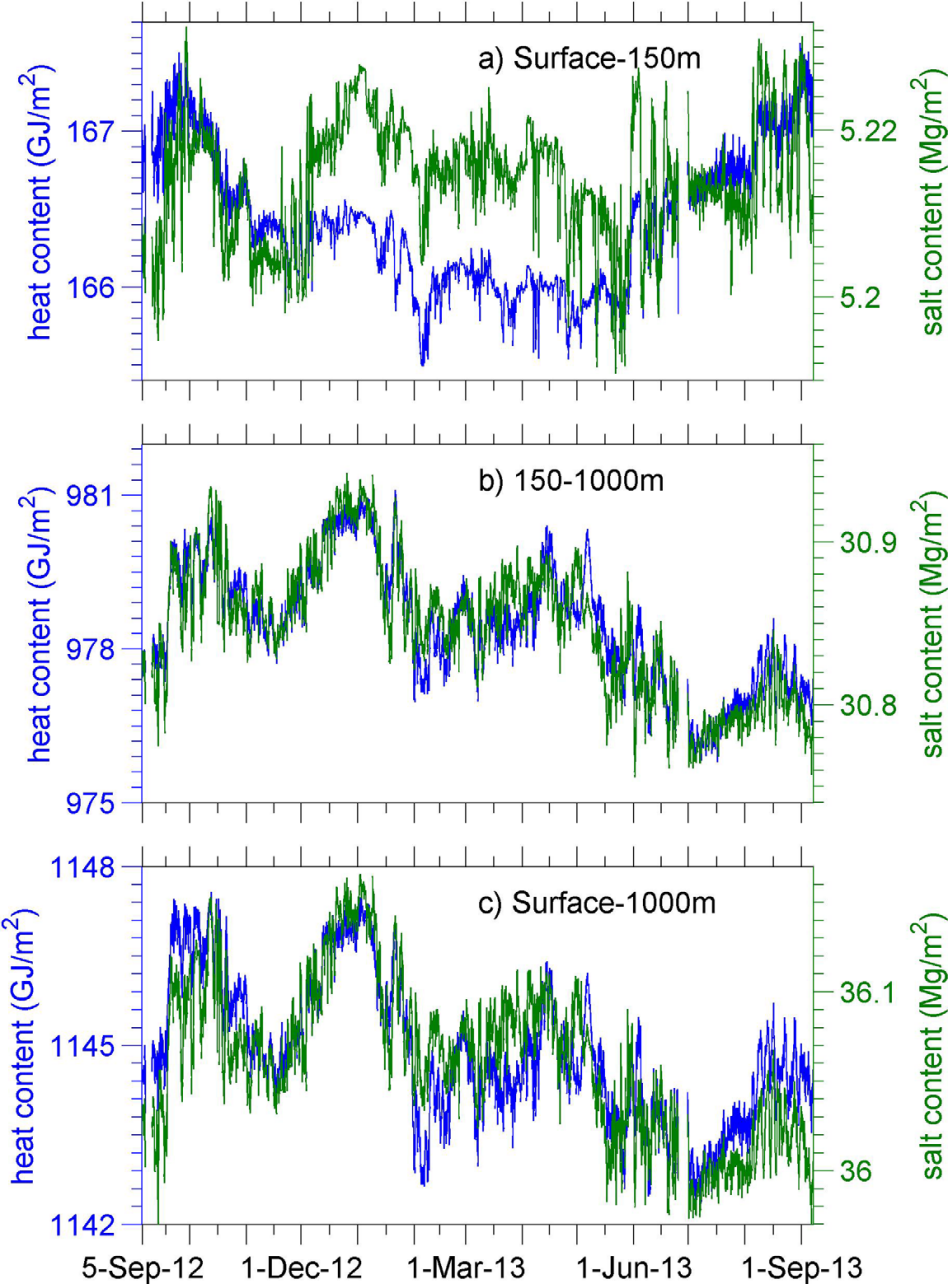
Time series of heat content (GJ m^−2^) (blue) and salt content (Mg m^−2^) summed over (a) the top 150 m, (b) 150–000 m, and (c) the top 1000 m.

In the OSMOSIS study area, the heat and salt content of the upper 1000 m are not dominated by the highly variable top 150 m. While the temperature varies over a much greater range in the uppermost 150 m than at depth (Figure 2e), salinity does not. Moreover, below 150 m the low‐pass filtered temperature and salinity show a dominant barotropic structure and also vary in phase with each other (Figure 2). Due to this largely barotropic structure, the variability in the heat and salt content of the upper 1000 m are in fact dominated by the variability below 150 m (Figure 9). The heat and salt content of the upper 1000 m are strongly correlated with each other (*r* = 0.73); this is largely due to the strong correlation below 150 m (*r* = 0.86, Figure 9).

*Prieto et al*. [2013] observed intermediate waters that were warmer and saltier in winter than summer between 2003 and 2010 along a section at 43°N extending 200 nm off Cape Finisterre. Similar seasonal cycles were observed in various locations in the Eastern North Atlantic by *Bray* [1982]; *Chidichimo et al*. [2010]; *Machin et al*. [2010] and are thought to be due to an increased admixture of cooler and fresher Western North Atlantic Water/Sub Arctic Intermediate Water during the summer. The results of *Bray* [1982] are based on CTD profiles at approximately 3 month intervals, those of *Prieto et al*. [2013] on CTD profiles at approximately 6 month intervals, those of *Machin et al*. [2010] on only 2 depths on a single mooring, and those of *Chidichimo et al*. [2010] on a composite profile obtained by combining observations from a moored array spread over approximately 1250 km. Our glider‐based observations, with full temporal coverage and resolution alongside good vertical resolution, provide new insights into the intraseasonal variability at all depths to 1000 m.

Below 150 m, we observe significant intraseasonal variability in temperature and salinity on a timescale of about 3 months (Figures 2, 5, and 9b). The studies described above [*Bray*, 1982; *Chidichimo et al*., 2010; *Prieto et al*., 2013] were unable to resolve this intraseasonal variability and therefore ascribed it to a seasonal cycle. The variability of the heat and salt content of the upper 1000 m (Figure 9c) is dominated by gyre‐scale and/or mesoscale variability below 150 m and not by the surface forcing. We suggest that other processes such as latitudinal variability in wind stress curl should be investigated using numerical models. The intraseasonal variability in the heat and salt content is not obviously related to the state of the North Atlantic Oscillation [*Barnston and Livezey*, 1987], the RAPID MOC volume transport time series [*Smeed et al*., 2015] or the local wind stress (supporting information Figure S2). This intraseasonal variability is also seen in the dissolved oxygen content below 700 m (not shown). Above 700 m, oxygen concentration variations are uncoupled from the intraseasonal variability in temperature and salinity, presumably due to horizontal or vertical entrainment of water with elevated oxygen concentrations which developed when the water was closer to the surface.

**Figure 10:**
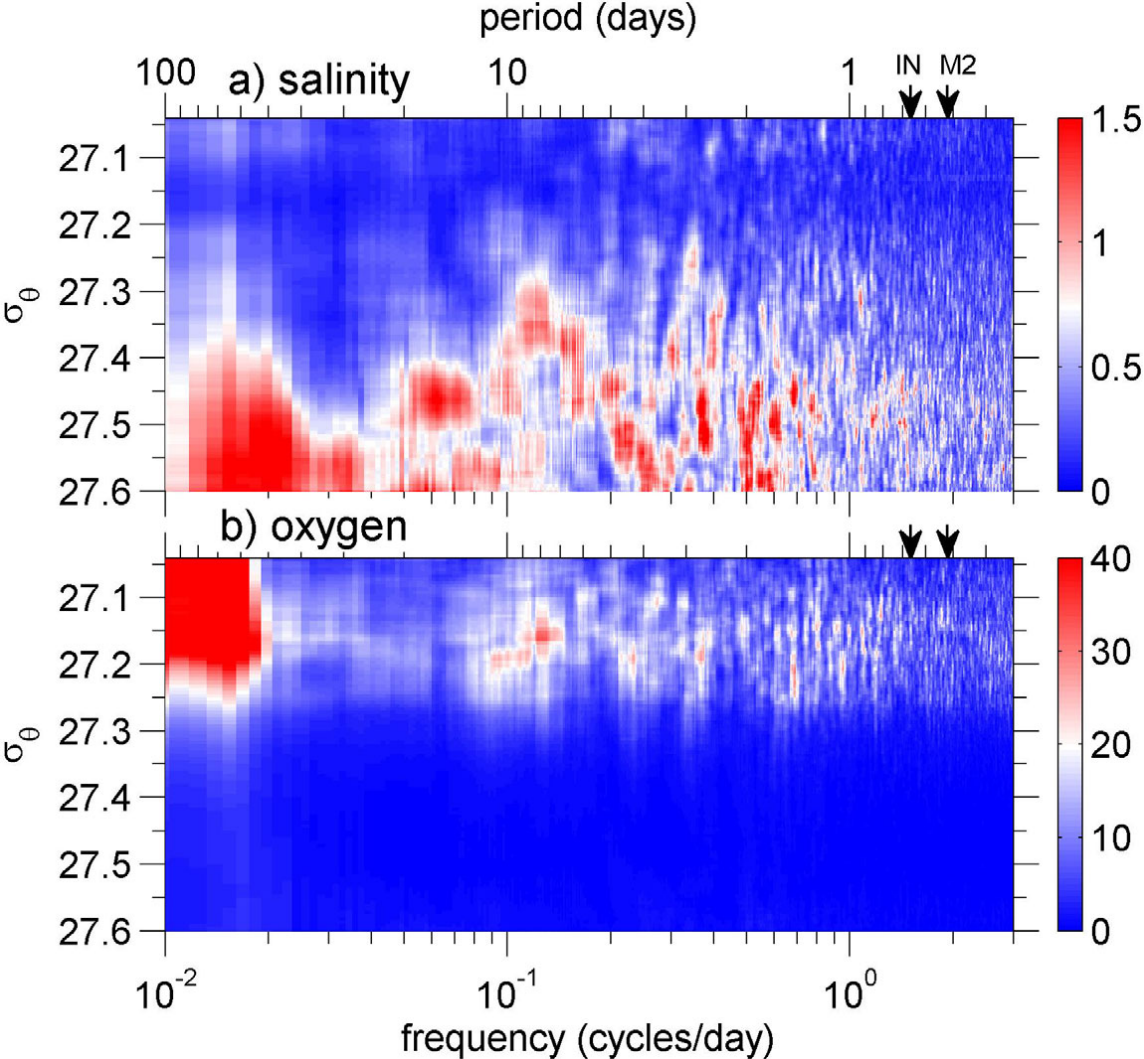
Variance preserving spectra for (a) salinity ( $\times 10^{-3}$) and (b) dissolved oxygen concentration calculated on density surfaces. In each plot, the colors show the power spectral density × frequency. The inertial frequency (IN) and M2 tidal frequency are marked as black arrows on the upper axis. Spectra are shown for $\sigma_{\theta }$ in the range 27.04–27.6. This is the range over which we have data for the complete year, as shown by the density contours on Figure 2.

A peak in variability at intraseasonal periods (40–100 days) could be due to Rossby waves [*Price and Rossby*, 1982]. This peak is still present in spectra calculated on density levels instead of depth (Figure 10) so Rossby waves are unlikely as the source of this variability since they would cause variability by heaving of the density surfaces. The intraseasonal variability is much more pronounced in the zonal component than the meridional component of the dive‐average currents, which might be associated with drifting quasi‐zonal jets [*van Sebille et al*., 2011].

### 3.4 Tidal and Inertial Frequencies

The spectra of the dive average currents show a very large peak in energy precisely at the M2 semidiurnal tidal frequency (Figures 5d and 5e). There is no evidence of significant variability at diurnal tidal frequencies. This semidiurnal signal is also noticeable in temperature (Figure 5a), where its energy increases with depth. This is present in salinity and oxygen but is less pronounced (Figures 5b and 5c). A barotropic tide would produce the greatest spectral energy where horizontal property gradients are greatest, near the surface. Since the tidal signal observed here increases with depth, we ascribe this to an internal tide. The OSMOSIS site lies in an abyssal plain far from the continental slope where internal tides are generated (∼ 350 km to the nearest point of the continental slope), so we would expect predominantly mode‐1 internal tides since higher modes would have dissipated before reaching the OSMOSIS site. Predicted mode‐1 isopycnal displacement (based on full‐depth profiles of the buoyancy frequency from OSMOSIS CTD surveys) would increase from zero at the surface to a maximum at depth of 1750 m, consistent with the glider‐based observations of an increased tidal signal from the surface to 1000 m. This peak is not visible in power spectra calculated on density surfaces (Figure 10), supporting the hypothesis that it is an internal tide. Spectra computed from moored instruments deployed between 50 m and 500 m depth as part of OSMOSIS also show a significant peak at the M2 tidal frequency (supporting information Figure S1).

At the inertial frequency ( $1.1\times 10^{-4}$ rad s^−1^, a period of 15.9 h), spectra of both the zonal and meridional dive‐average velocities show a small increase in energy (Figures 5d and 5e). The upper 100 m also displays slightly increased energy at near‐inertial frequencies in both temperature and density. This is consistent with wind‐generated near‐inertial waves propagating downward [*Pollard*, 1970; *Alford*, 2001, 2003].

## 4 Conclusions

A year‐long time series of temperature, salinity and dissolved oxygen concentration at 2 hourly intervals in the uppermost 1000 m of the ocean was obtained from gliders. This provides an ideal data set for validation of process or regional models. The ENACW shows evidence of warming since the 1950s/1960s. There is a strong seasonal cycle in near‐surface temperature and mixed layer depth, as expected, consistent with net surface heat flux. The shoaling of the ML in spring is intermittent and interspersed with deepening events. Variations in ML salinity are not explained by local freshwater fluxes and must therefore be influenced by horizontal advection of different water masses associated with changes in local gyre‐scale circulation, and/or mesoscale eddies. A strong peak in variability is observed at the M2 tidal frequency due to a mode‐1 baroclinic internal tide. In terms of mixing processes, the ENACW is susceptible to salt fingering for much of the year. Gravitational instabilities are seen in winter, associated with rapid deepening of the mixed layer. At about 700–900 m, the depth of the dissolved oxygen minimum, the water column is susceptible to diffusive layering, particularly when MW is present. The deep variability is dominated by the intermittent appearance of patches of MW, and this variability in temperature and salinity is present at all time scales due to the filamented nature of these patches. Below ∼150 m we see intraseasonal variability (on time periods of 2–5 months) which dominates the variability in heat and salt content variability in the entire upper 1000 m. The unique ability of ocean gliders to resolve both high vertical and temporal resolution highlights the importance of intraseasonal variability in upper ocean heat and salt content, variations that may be aliased by traditional observing techniques.

## Supporting information

Supporting Information S1

Figure S1

Figure S2
