# Supplementary material for: The vertical structure of upper ocean variability at the Porcupine Abyssal Plain during 2012–2013
Source: J Geophys Res Oceans. 2016 May 13;121(5):3075–89. doi: 10.1002/2015JC011423 (PMC5084758; doi:10.1002/2015JC011423)
Supplement: Supplementary file 1 — Supporting Information S1 [file JGRC-121-3075-s001.pdf]

Supporting Information for "The Vertical Structure of Upper Ocean Variability at the Porcupine Abyssal Plain during 2012-2013"

Gillian M. Damerell,[1] Karen J. Heywood,[1] Andrew F. Thompson,[2] Umberto Binetti,[1] Jan Kaiser,[1]

Contents of this file

1. Text S1
2. Figure S1
3. Text S2
4. Figure S2

Introduction

Corresponding author: Gillian Damerell, Centre for Ocean and Atmospheric Sciences, School of Environmental Sciences, University of East Anglia, Norwich, NR4 7TJ, UK. (g.damerell@uea.ac.uk)

[1]Centre for Ocean and Atmospheric Sciences, School of Environmental Sciences, University of East Anglia, Norwich, UK.

[2]California Institute of Technology, Pasadena, USA.

Text S1. Figure 1 shows some example spectra computed from moored instruments deployed as part of OSMOSIS. These instruments sampled every 10 minutes, so resolve the internal wave field which the gliders do not. Moreover, these instruments, being moored, did not move around the gliders' sampling box. Thus, spectra calculated from these instruments should not be subject to distortion of the observed spectra, relative to the true values, due to aliasing of the internal wave field and glider movement (as discussed in the main text). The spectra from the moored instruments are extremely similar to those from the gliders, lending confidence to the decision that these issues do not significantly influence the conclusions here.

Text S2. Figure 2 illustrates the lack of correlation between the intraseasonal variability in heat and salt content below 150 m, and the RAPID-MOC transport, local wind speed, and North Atlantic Oscillation index.

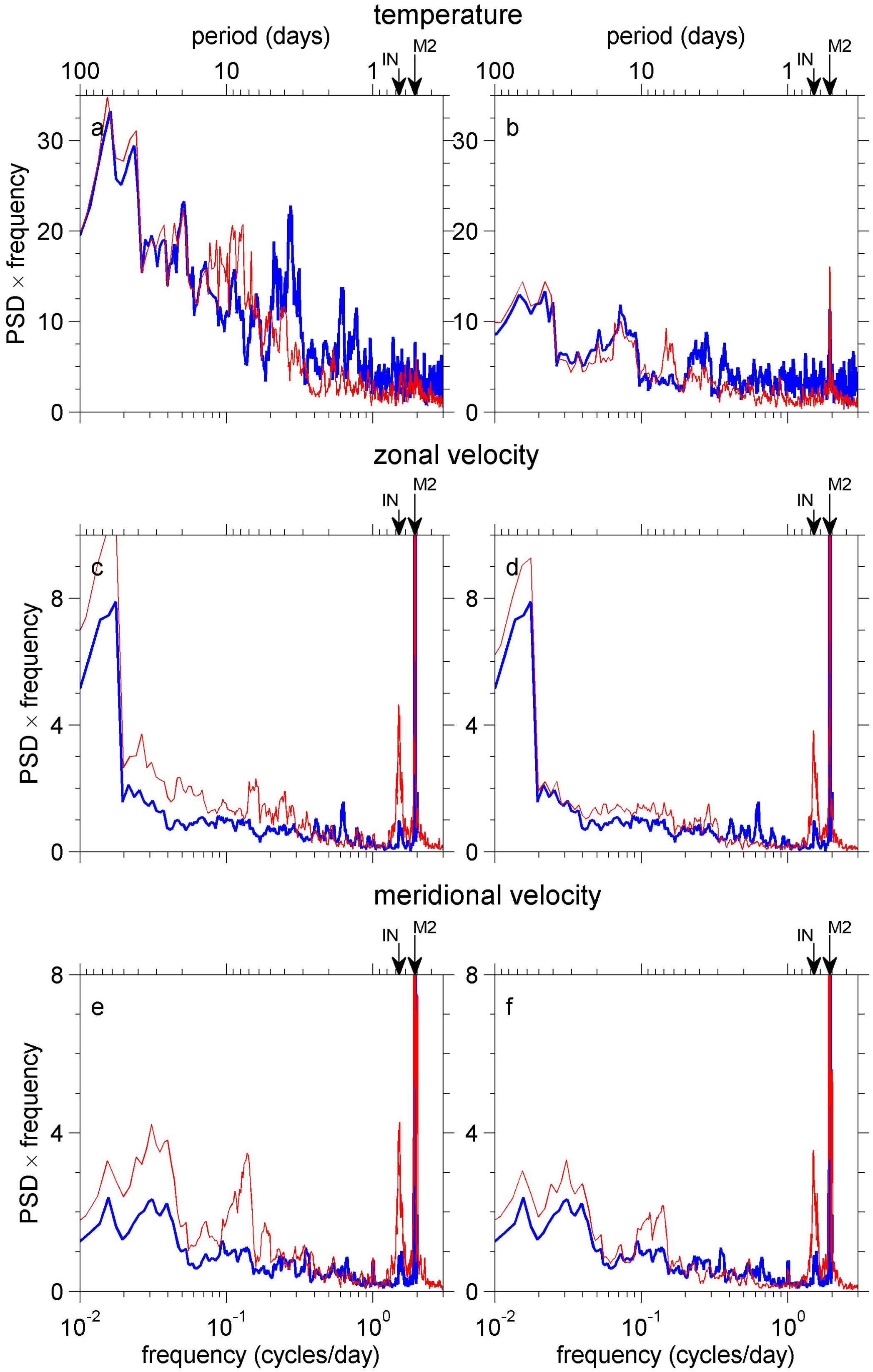
temperature
period (days)
100
10
1
IN
M2
a
b
PSD × frequency
zonal velocity
c
d
meridional velocity
e
f
frequency (cycles/day)

Figure 1. Variance preserving spectra for (top) temperature, (middle) zonal velocity and (bottom) meridional velocity. On each plot, spectra from instruments on the central mooring, located at 48.69°N, 16.19°W, are shown in red, at depths of (left column) 250 m and (right column) 450 m. On the top panels, the blue lines are spectra calculated from the glider-measured temperature at (left) 250 m and (right) 450 m. In the middle and bottom panels, the blue lines show the spectra of the zonal and meridional components, respectively, of the glider dive-average currents (same in both columns).

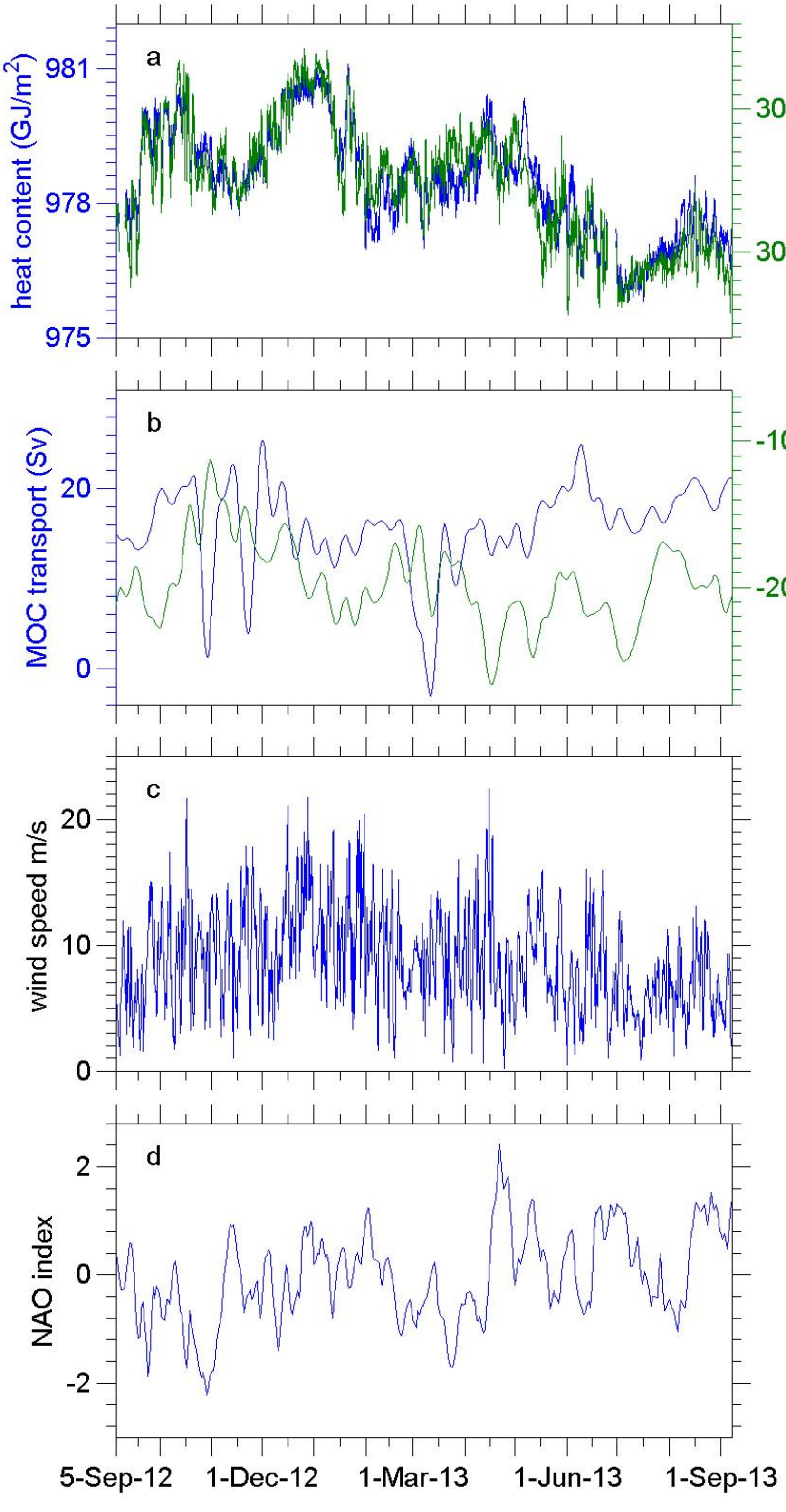

Figure 2. Time series of (a) heat (blue) and salt (green) content from 150 m to 1000 m, as in Figure 8b, (b) transport from the RAPID MOC array with total MOC transport in blue, and the thermocline recirculation (0-800 m) in green (c) wind speed from the ECMWF ERA-Interim dataset at the closest gridpoint to the OSMOSIS site and (d) North Atlantic Oscillation index.
