# Supplementary figures and images for: The vertical structure of upper ocean variability at the Porcupine Abyssal Plain during 2012–2013
Source: J Geophys Res Oceans. 2016 May 13;121(5):3075–89. doi: 10.1002/2015JC011423 (PMC5084758; doi:10.1002/2015JC011423)

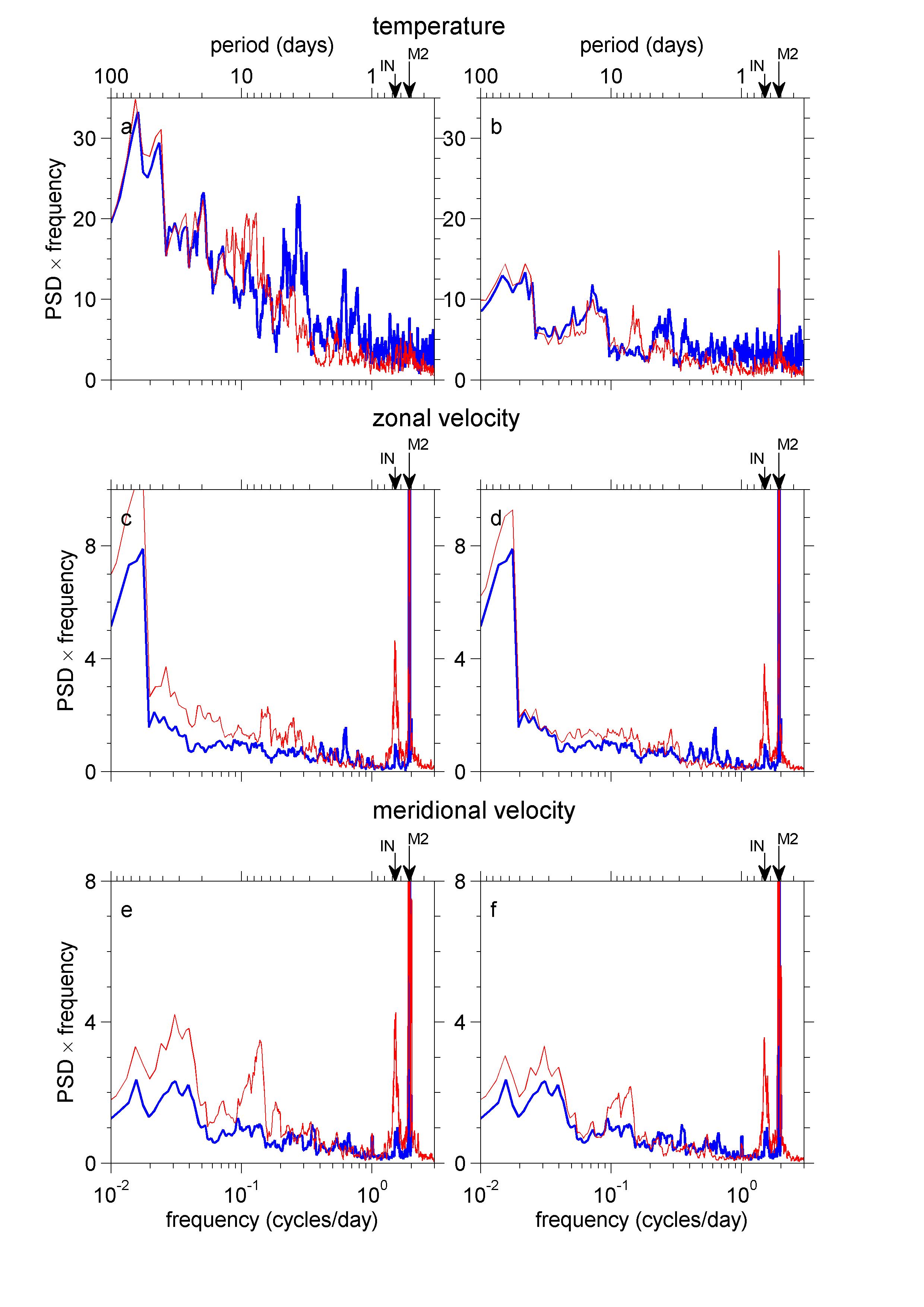

Supplement: Supplementary file 2 — Figure S1 [file JGRC-121-3075-s002.jpg]

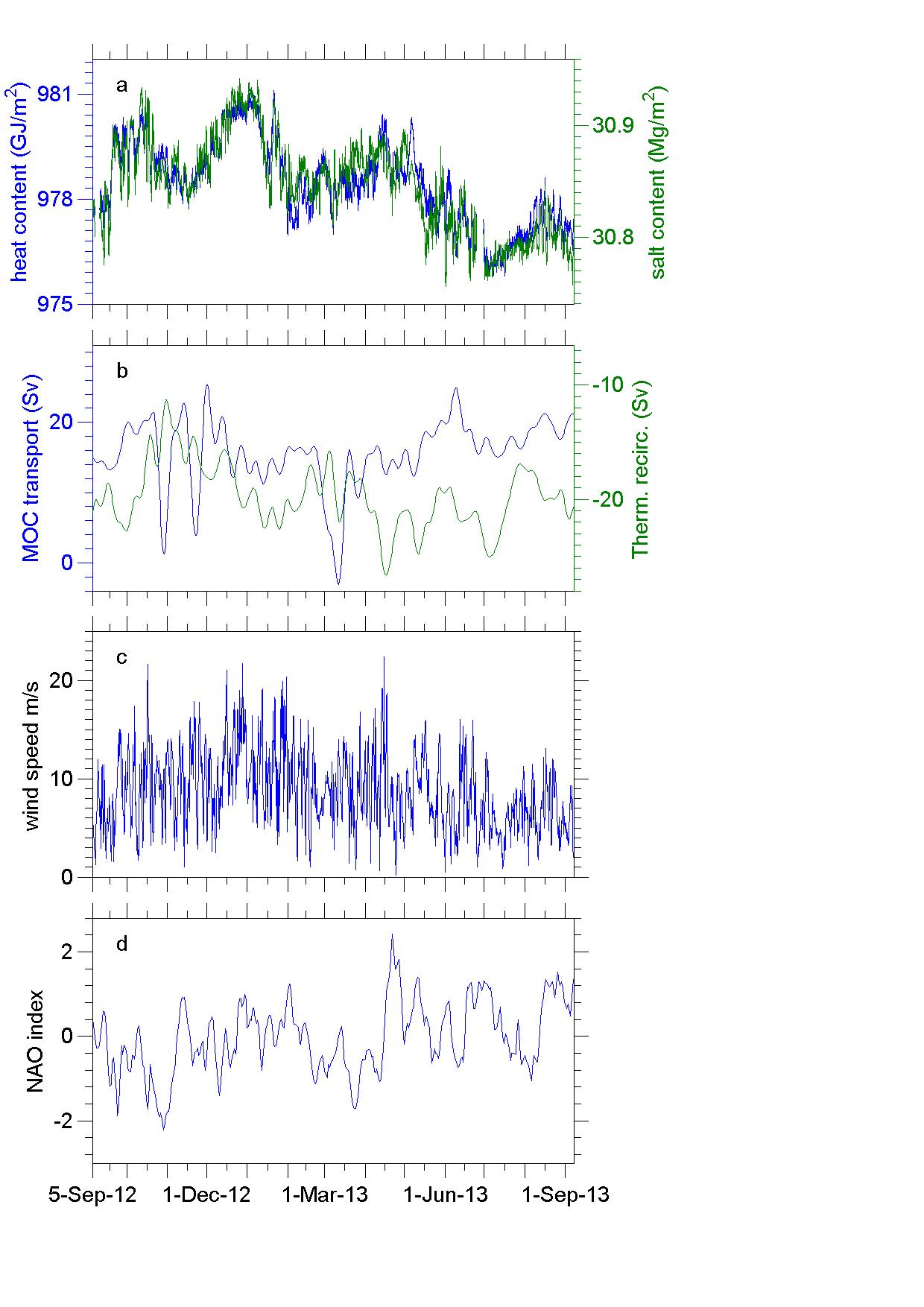

Supplement: Supplementary file 3 — Figure S2 [file JGRC-121-3075-s003.jpg]
